# The Effect of Colchicine on Atrial Fibrillation: A Systematic Review and Meta-Analysis

**DOI:** 10.7759/cureus.35120

**Published:** 2023-02-17

**Authors:** Sharath Kommu, Shalini Arepally

**Affiliations:** 1 Hospital Medicine, Marshfield Clinic Health System, Rice Lake, USA; 2 Family Medicine, Marshfield Clinic Health System, Rice Lake, USA

**Keywords:** coronary artery disease, post ablation, post-cardiac surgery, atrial fibrillation, colchicine

## Abstract

Colchicine is a potent anti-inflammatory agent whose benefits have been explored for various conditions, including atrial fibrillation (AF). In this article, we tried to understand why colchicine might be beneficial in AF and reviewed various studies that looked at the effect of colchicine against AF. We followed the PRISMA algorithm and undertook a literature search to identify studies with control groups that looked at the effect of colchicine against AF and conducted a meta-analysis. We identified six studies on post-cardiac surgical patients, three on post-pulmonary vein isolation (PVI)/ablation patients, and two on coronary artery disease. In patients who underwent cardiac surgery, we found that colchicine is beneficial against postoperative atrial fibrillation (POAF) with a relative risk (RR) of 0.70 (95% CI of 0.58 to 0.84) and a p-value of 0.0001. We also found that in patients who underwent PVI/ablation, colchicine is beneficial in decreasing AF recurrence over three months with an RR of 0.57 (95% CI of 0.39 to 0.83) and a p-value of 0.0032 and over 12 months follow-up with an RR of 0.58 (95% CI of 0.42 - 0.80) and a p-value of 0.0008. Our meta-analysis showed that in patients with coronary artery disease, colchicine had no significant benefit in decreasing the incidence of AF with a hazard ratio (HR) of 0.86 (95% CI of 0.69 - 1.06) and a p-value of 0.16. From this study, we conclude that colchicine may be beneficial for decreasing the incidence of AF in post-cardiac surgery patients and post-PVI/ablation patients. It may not decrease the incidence of AF in patients with coronary artery disease.

## Introduction and background

Colchicine has potent anti-inflammatory effects, and various studies have examined its benefits in different cardiac conditions. For example, the Investigation on Colchicine for Acute Pericarditis (ICAP) [1] and the COlchicine for acute PEricarditis (COPE) [2] trial have shown colchicine's benefit for the treatment of pericarditis and the 2015 European Society of Cardiology (ESC) guidelines for the diagnosis and management of pericardial diseases have recommended colchicine for the treatment of acute pericarditis [3]. The benefits of colchicine are also being explored in other cardiac conditions, including coronary artery disease (CAD) and atrial fibrillation (AF). In this article, we attempted to review why colchicine might be beneficial in AF, the various studies on colchicine that address its effect on AF, and conducted a meta-analysis.

Risk factors for AF include hypertension, CAD, valvular heart disease, congenital heart disease, obstructive sleep apnea, type 2 diabetes mellitus, hyperthyroidism, and genetic factors. Inflammation is also identified as one of the risk factors for AF. Various observational studies have reported that elevated serum levels of C-reactive protein (CRP), a marker of inflammation, are associated with the development of AF [4], history of atrial arrhythmias [5], and the development of AF after cardiac surgery [6, 7]. Inflammatory activation operates in conjunction with oxidative stress to create a pathway promoting atrial electrical and structural remodeling and, thus, atrial ectopy, interstitial fibrosis, and atrial arrhythmias [8]. Various agents with anti-inflammatory properties, including corticosteroids, statins, and colchicine, have been studied for their effect on AF. The most studied mechanism of action of colchicine is its anti-inflammatory effect, wherein it disrupts cytoskeletal functions. It inhibits the polymerization of β-tubulin into microtubules and prevents the activation, degranulation, and migration of neutrophils. Colchicine may also interfere with the intercellular assembly of the inflammasome complex in neutrophils and monocytes that mediate the activation of interleukin-1β.

Numerous animal studies have examined the role of inflammation in AF and the potential benefit of colchicine. Wu et al. found that colchicine prevents AF in rats by inhibiting interleukin (IL)-1β-induced IL-6 release and subsequent atrial fibrosis [9]. Singhal et al. studied the failing heart of rabbits. They found that colchicine regulates ion channel gene expression and activates the PI3K/AKT/eNOS signaling pathway in rabbits with heart failure, which may reverse atrial remodeling and suppress AF [10]. Yao et al. found NLRP3 inflammasome activity contributing to increased premature atrial contractions and atrial fibrillation [11]. They found that NLRP3 inflammasome activity was increased in the atrial cardiomyocytes of patients with paroxysmal AF and chronic AF. The mouse model with active NLRP3 inflammasome activity developed spontaneous premature atrial contractions and inducible AF, which was attenuated by a specific NLRP3 inflammasome inhibitor. Though colchicine is known to act on the inflammasome complex in neutrophils and monocytes, it is unclear if it has a similar role on the inflammasome of cardiac myocytes.

Injury or exposure to a pathogen can trigger an inflammatory response [12]. Like any other organ in the body, an insult to the heart, either in the form of cardiac surgery or coronary vascular event, can cause a local inflammatory response. In certain instances, mainly if there are other associated risk factors, this inflammatory response can trigger AF. Understanding how inflammation contributes to AF, we look at the anti-inflammatory benefits of colchicine, as have appeared in various studies examining several cardiac conditions. This meta-analysis, examining each of several cardiac conditions, will help express colchicine's potential benefit, if any, when employed against AF.

## Review

Materials and Methods

The current meta-analysis is conducted as per the guidelines of Preferred Reporting Items for Systematic Review and Meta-analyses (PRISMA).

Search Strategy and Study Selection

We searched the online databases PubMed and EMBASE using the keywords "colchicine" and "atrial fibrillation". We selected the articles published between January 2010 and December 2022. Studies were included in our meta-analysis if the following criteria were met: 1) clinical studies on humans that include a comparison (control) group; 2) study population included those with a cardiac condition; 3) involved the use of colchicine; and 4) atrial fibrillation is the endpoint or one of the endpoints of the study. All titles and abstracts of the results of our computerized search were reviewed by both authors independently for potential inclusion in our study. In addition to our computerized search, we manually reviewed the reference list of all retrieved articles to complete our search. We resolved any disagreements through mutual discussion.

Data Extraction

After identifying the relevant articles, we extracted the data relevant to our meta-analysis, including the name of the study/trial, name of the authors, the year the study was published, study population, the number of patients included in the experimental and in the placebo group, number of events of atrial fibrillation under each group and the outcome or result of the study in relation to atrial fibrillation.

Statistical Analysis

Data were analyzed using the software R version 4.0.4 (R Foundation, Vienna, Austria) and RStudio version 1.4.1103 (Posit PBC, Boston, Massachusetts), including the package "meta" [13]. Results included both the common or fixed effects model and the random effects model using the DerSimonian-Laird estimator. Two measures of heterogeneity were calculated - Higgins & Thompson's I^2^ Statistic and Heterogeneity Variance τ^2^, commonly used for assessing the need for random effects. A p-value of <0.1 was considered significant for heterogeneity, supporting the use of the random effects model. Based on the observed number of events of atrial fibrillation in both arms of each study, relative risk (RR), 95% confidence intervals (CI), and p-values were calculated using standard, large-sample approximations. For studies that presented hazard ratios (HR), event numbers and associated statistics were approximated from estimated event rates. A calculated p-value of <0.05 was considered significant evidence of treatment differences.

Results

**Figure 1:**
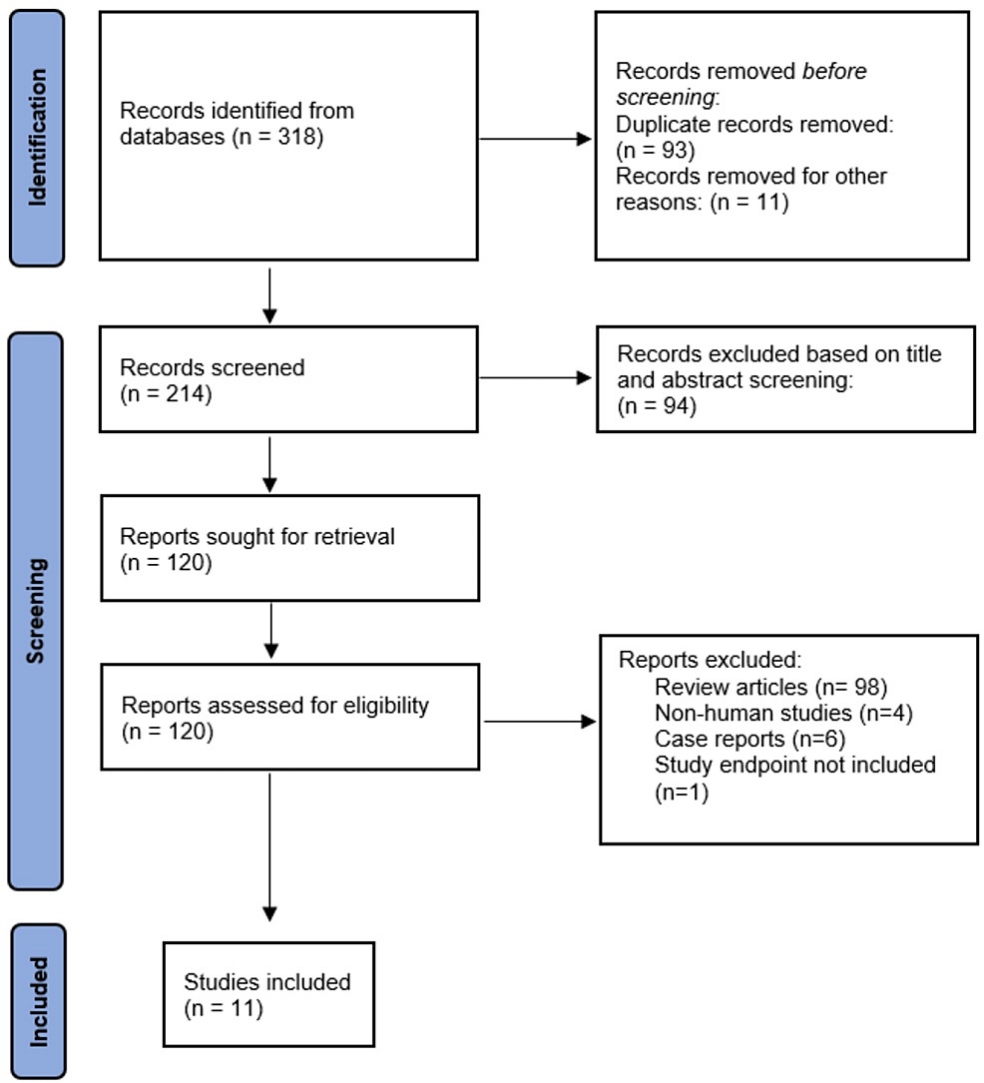
PRISMA flow chart depicting the selection of studies PRISMA - Preferred Reporting Items for Systematic Review and Meta-Analyses

Our online database search yielded 318 references. After further review and identifying duplicates (93), other exclusions (11), and excluding records based on the title and abstract screening (94), we sought 120 reports for retrieval. After further exclusions, we identified a total of 11 studies that can be included in our meta-analysis (Figure 1). Out of these, six were relevant studies on post-cardiac surgical patients (Table 1), three on post-pulmonary vein isolation (PVI)/ablation patients (Table 2), and two on patients with coronary artery disease (Table 3), all of which studied colchicine, with AF being the endpoint or one of the endpoints.

**Table 1:** The studies on the effect of colchicine on atrial fibrillation in post-cardiac surgery patients *- likelihood ratio Chi-squared p-value, RR - relative risk, CI - confidence interval

Study	Year	Study population	No. of patients	No. of events	RR (95% CI)	p-value*	Result
Imazio et al., COPPS trial [14]	2011	Cardiac surgery	Colchicine - 169, placebo - 167	Colchicine - 20, placebo - 37	0.53 (0.32-0.87)	0.011	Positive
Imazio et al., COPPS 2 [15]	2014	Cardiac surgery	Colchicine - 180, placebo - 180	Colchicine - 61, placebo - 75	0.81 (0.62-1.06)	0.128	Neutral
Tabbalat et al., END-AF trial [16]	2016	Cardiac surgery	Colchicine - 179, no colchicine -181	Colchicine - 26, placebo - 37	0.71 (0.45-1.12)	0.139	Neutral
Zarpelon et al. [17]	2016	Cardiac surgery	Colchicine - 71, placebo - 69	Colchicine - 5, placebo - 9	0.54 (0.19-1.53)	0.234	Neutral
Tabbalat et al., END-AFLD [18]	2020	Cardiac surgery	Colchicine - 81, placebo - 71	Colchicine - 13, placebo - 13	0.88 (0.44-1.76)	0.712	Neutral
Shvartz et al., COCS Trial [19]	2022	Cardiac surgery, Aortic valve repair	Colchicine - 113, placebo - 127	Colchicine - 21, placebo - 39	0.61 (0.38-0.96)	0.029	Positive

**Table 2:** The studies on the effect of colchicine on atrial fibrillation in post-pulmonary vein isolation (PVI)/ablation patients *- likelihood ratio Chi-squared p-value, RR - relative risk, CI - confidence interval, PVI - pulmonary vein isolation

Study	Year	Study population	No. of patients	Study duration	No. of events	RR (95% CI)	p-value*	Result
Defteros et al. [20]	2012	PVI	Colchicine - 81, placebo - 80	3 months	Colchicine - 13, placebo - 27	RR 0.48 (0.26-0.85)	0.009	Positive
Egami et al. [21]	2015	Ablation	Including both smaller LA-EAT group (n=62) and larger LA-EAT group (n=60) colchicine - 42, non-colchicine - 80	3 months	Colchicine - 14, placebo - 40	RR 0.67 (0.40-1.05)	0.076	Neutral
				12 months	Colchicine - 7, placebo - 29	RR 0.46 (0.22-0.91)	0.020	Positive
Defteros et al. [22]	2014	Ablation	Colchicine - 103, Placebo - 103	12 months	Colchicine - 32, placebo - 51	RR 0.63 (0.44-0.88)	0.007	Positive

**Table 3:** The studies on the effect of colchicine on atrial fibrillation in patients with coronary artery disease **- approximate large-sample p-value based on the width of confidence interval, HR - hazard ratio, CI - confidence interval

Study	Year	No. of patients	Study population	Endpoint	No. of events	HR (95% CI)	p-value**	Result
Tardif et al. COLCOT [23]	2019	Colchicine - 2366, placebo - 2379	Post myocardial infarction	Atrial fibrillation	Colchicine - 6, placebo - 40	0.93 (0.59-1.46)	0.754	Neutral
Nidorf et al., LoDoCo2 [24]	2020	Colchicine - 2762, placebo - 2760	Chronic coronary artery disease	New-onset or first recurrence of atrial fibrillation or atrial flutter	Colchicine - 126, placebo - 148	0.84 (0.66-1.07)	0.157	Neutral

Post-Cardiac Surgery

AF occurs in 15 to 40 percent of patients in the early postoperative period following coronary artery bypass graft surgery (CABG) [25-28]. In the 2011 COPPS trial by Imazio et al. [14], treatment with either placebo or colchicine began on postoperative day three. Colchicine was given in a 1 mg twice-daily dose for the first day, followed by a maintenance dosage of 0.5 mg twice daily for one month in patients weighing 70 kg or more. Halved doses were given to patients weighing less than 70 kg or who were intolerant to the highest dose. The colchicine group had a lower incidence of postoperative atrial fibrillation (POAF) (12.0% versus 22.0%, respectively; p=0.021; relative risk reduction, 45%) compared to placebo [14]. This study also showed a shorter duration of POAF and shorter in-hospital stay in the colchicine group [14]. This study thus mainly showed the beneficial effects of colchicine against AF in post-cardiac surgical patients.

The outcomes of the 2011 COPPS trial were somewhat limited in scope because colchicine had been administered on the third postoperative day. Thus, another study, the 2014 COPPS 2 trial, was conducted by Imazio et al. [15]. In this study, colchicine was started before surgery to see if the benefits could be further optimized. It was a randomized control trial, and patients were given either colchicine or Placebo beginning 48 and 72 hours before surgery and continued for one month. AF occurred in 33.9% of patients in the colchicine group and 41.7% of patients in the placebo group, with an absolute difference of 7.8% and 95% CI of -2.2% to 17.6% [15]. This study did not show additional benefits with colchicine against POAF in patients who underwent cardiac surgery. 

In the 2016 END-AF study by Tabbalat et al., [16] colchicine was administered at a dose of 2 mg given 12 to 24 hours before surgery and 1 mg given 4 hours before or immediately after surgery and then continued at a dose of 0.5 mg twice daily until hospital discharge. Half the dose was given to patients weighing less than 70 kg or intolerant to the full dose. The primary endpoint of AF occurred in 14.5% of the colchicine group and 20.5% in the no-colchicine group (relative risk reduction of 29.3% and p-value of 0.14) [16]. Diarrhea occurred in 24.6% of the colchicine group and 5.5% of the no-colchicine group (p<.001) [16]. Diarrhea led to the discontinuation of colchicine in 23 (52%) of the 44 patients [16]. This study showed that in patients who underwent cardiac surgery, colchicine was not beneficial in reducing the incidence of early POAF (before hospital discharge). This study also showed that diarrhea is a significant complication that occurred in 24.6% of patients in the colchicine group.

In the 2016 study by Zarpelon et al., [17] one group received oral colchicine at the dose of 1 mg twice daily, 24 hours before surgery, followed by 0.5 mg twice daily until hospital discharge. There was no significant decrease in the incidence of AF compared to control group patients (7.04% versus 13.04%, respectively; p=0.271) [17]. This study also showed no significant benefit from the death of any cause or length of hospital stay from colchicine use. 

In another study by Tabbalat et al. called END-AF low dose [18], low-dose colchicine was used at 1 mg administered 12-24 hours before cardiac surgery, followed by 0.5 mg daily until discharge. In the colchicine group, AF occurred in 16% versus 18.3% in the placebo group, with an odds ratio (OR) of 0.85 (95% CI of 0.37-1.99) [18]. In this study, the investigators found that in patients undergoing cardiac surgery, though low-dose colchicine was well tolerated, it did not prevent POAF. 

The 2022 COCS trial study by Shvartz et al. [19] is one of the latest randomized controlled trials investigating the relationship between colchicine and AF. Patients undergoing cardiac surgery or aortic valve replacement were randomized to receive either 1 mg of colchicine or placebo 24 hours before surgery and on days two, three, four, and five. Patients were monitored until discharge from the hospital for POAF. The colchicine group developed POAF in 18.6% of cases, while 30.7% developed it in the control group (OR 0.515; 95% CI 0.281-0.943; p=0.029) [19], thus confirming the effectiveness of colchicine in preventing POAF after cardiac surgery/aortic valve replacement.

**Figure 2:**
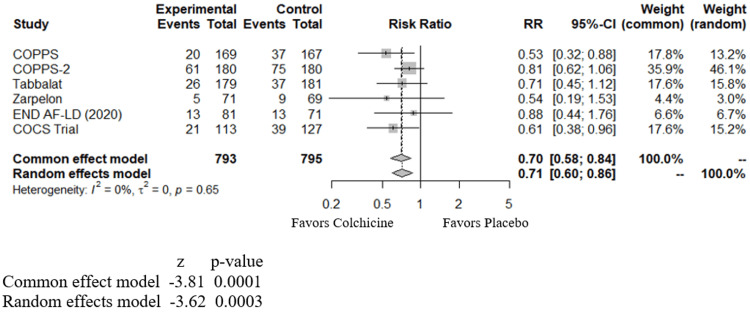
Forest plot showing the effect of colchicine on atrial fibrillation in post-cardiac surgical patients RR - relative risk; CI - confidence interval; COPPS - study by Imazio et al., 2011 [14]; COPPS2 - study by Imazio et al., 2014 [15]; Tabbalat - study by Tabbalat et al., 2016 [16]; Zarpelon - study by Zarpelon et al., 2016 [17]; END AF-LD - study by Tabbalat et al., 2020 [18]; COCS Trial - study by Shvartz et al., 2022 [19]

Interestingly, of the above six studies, only two showed statistically significant benefits in reducing AF in post-cardiac surgical patients. The remaining four showed a neutral effect. Including all the above studies, our meta-analysis (Figure 2) showed a relative risk (RR) of 0.70 with a 95% CI of 0.58 to 0.84 and a p-value of 0.0001, indicating that colchicine is beneficial in reducing POAF after cardiac surgery.

Post-PVI/Ablation

Ablation therapy can initiate a proinflammatory process that is implicated in early AF recurrence [29]. A study by Deftereos et al. [20] published in 2012 was a randomized trial that looked at the effects of colchicine in post-PVI/ablation cases; subjects received colchicine or a placebo for three months. The colchicine group received 0.5 mg twice daily of oral colchicine from the day of ablation. In the three-month follow-up, 33.5% of patients in the placebo group and 16% of patients in the colchicine group had a recurrence of AF, with an odds ratio (OR) of 0.38 (95% CI: 0.18-0.80) [20]. This study showed the benefit of colchicine in post-PVI/ablation patients at three months.

In another study by Deftereos et al. [22] published in 2014, patients were randomized to receive colchicine (at a dose of 0.5 mg twice daily) or a placebo for three months from the day of the ablation procedure. The colchicine group had AF recurrence at 31.1% (32/103) and the control group at 49.5% (51/103) during the 12-month follow-up, with a p-value of 0.010 [22]. This study showed the benefit of colchicine in post-PVI/ablation patients at 12 months.

The epicardial adipose tissue (EAT) has been shown to release activated proinflammatory cytokines and to associate with the development of AF [30, 31]. A 2015 study by Egami et al. [21] studied the effect of colchicine in post-AF ablation patients in relation to left atrial epicardial adipose tissue (LA-EAT). Patients were divided into a larger LA-EAT group and a smaller LA-EAT group according to the median of LA-EAT volume of 22.4 cm3. The colchicine group received 0.5 mg/day for two weeks after AF ablation, and the incidence of AF was studied in three- and 12-month periods in both the colchicine and non-colchicine group. This study found that in the colchicine group with larger LA-EAT, the incidence of AF recurrence was lower in 12 months after ablation when compared to the non-colchicine group (10.5% vs. 34.2%, p=0.06) [21]. However, in the smaller LA-EAT group, the incidence of AF recurrence was similar between the colchicine group and non-colchicine groups. This study concluded that in patients with AF ablation, colchicine might reduce AF recurrence in patients with larger LA-EAT volumes.

**Figure 3:**
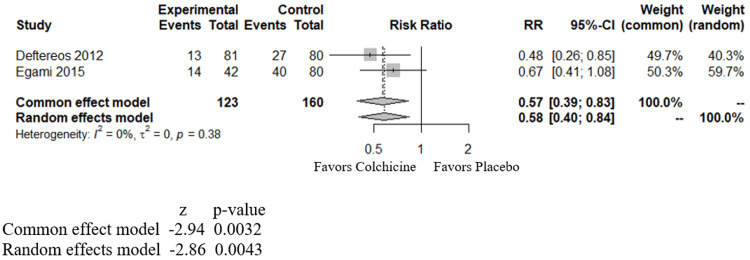
Forest plot showing the effect of colchicine on atrial fibrillation in post-pulmonary vein isolation (PVI)/ablation patients over three months RR - relative risk; CI - confidence interval; Deftereos - study by Deftereos et al., 2012 [20]; Egami - study by Egami et al., 2015 [21].

**Figure 4:**
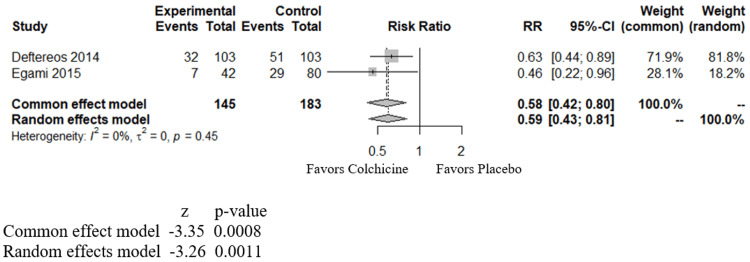
Forest plot showing the effect of colchicine on atrial fibrillation in post-pulmonary vein isolation (PVI)/ablation patients over 12 months RR - relative risk; CI - confidence interval; Deftereos - study by Deftereos et al., 2014 [22]; Egami - dtudy by Egami et al., 2015 [21]

LA-EAT measurements are not part of routine workup; hence, for ease of analysis in our study, we clubbed the LA-EAT groups into one group to analyze the effect of colchicine on AF in post-AF ablation cases. In our study, we included the incidence of AF recurrence in three months analyzed in the study by Deftereos et al. (2012) and Egami et al. (2015) in one sub-group (Figure 3) and AF recurrence in 12 months from the study by Deftereos et al. (2014) and Egami et al. (2015) in another sub-group (Figure 4). We found that over three months, colchicine was beneficial in decreasing AF recurrence with an RR of 0.57 (CI of 0.39 to 0.83) and a p-value of 0.0032 (Figure 3). Over 12 months, colchicine was again beneficial, with an RR of 0.58 (CI of 0.42 - 0.80), with a p-value of 0.0008 (Figure 4).

Post CAD

**Figure 5:**
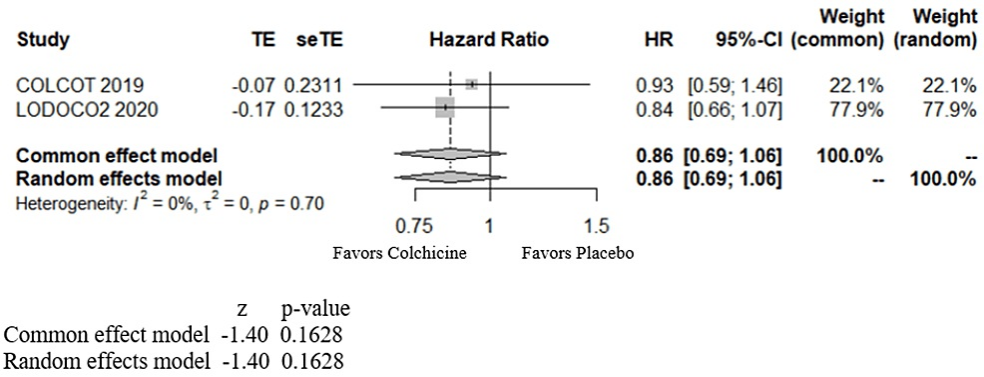
Forest plot showing the effect of colchicine on atrial fibrillation in coronary artery disease patients HR - hazard ratio; CI - confidence interval; COLCOT - study by Tardif et al., 2019 [23]; LODOCO2 - study by Nidorf et al., 2020 [24]

As inflammation plays an essential part in atherosclerosis, exploring the role of colchicine in coronary artery disease is important. The LoDoCo [32], COLCOT [23], and LoDoCo2 [24] trials looked at this role. Of these three studies, only the COLCOT and LoDoCo2 trials included a sub-analysis of AF incidence. The COLCOT study evaluated the effects of colchicine on cardiovascular outcomes in patients who recently had myocardial infarction (MI), finding that among patients with a recent myocardial infarction, colchicine at a 0.5 mg daily dose led to a significantly lower risk of ischemic cardiovascular events than the placebo. AF is one of the exploratory endpoints in the COLCOT study. Six patients (1.5%) in the colchicine group and 40 patients (1.7%) in the placebo group developed AF with a Hazard ratio (HR) of 0.93 (95% CI 0.59-1.46) [23]. Hence, this study showed no benefit of colchicine use for recent MI patients in preventing AF. The LoDoCo2 trial published in 2020 examined patients with chronic coronary artery disease and found that the risk of cardiovascular events was significantly lower among those who received 0.5 mg of colchicine once daily than among those who received the placebo. One of this study's additional endpoints was the new onset or first recurrence of AF, which was noted in 126 (4.6%) patients in the colchicine group and 148 (5.4%) in the control group with HR of 0.84 (95% CI 0.66-1.07) [24]. This study likewise shows no beneficial effect of colchicine on AF in patients with coronary artery disease. We analyzed the AF endpoint in patients with coronary artery disease from COLCOT and LoDoCo2 trials. Our meta-analysis shows that in patients with coronary artery disease, colchicine had no significant benefit in decreasing the incidence of AF with an HR of 0.86 (95% CI of 0.69 - 1.06) with a p-value of 0.16 (Figure 5).

Discussion

The studies, as indicated above (Table 1 and Table 2), have shown conflicting evidence on the benefits of colchicine in POAF and mostly beneficial effects in post-PVI/ablation cases. To date, the literature on colchicine's effect on POAF and post-ablation AF has included various meta-analyses looking at their effects both together and separately [33-38].

Some of the meta-analyses that studied POAF and post-cardiac ablation/PVI together include those by Trivedi et al. in 2014 [33], Verma et al. in 2015 [34], Papageorgiou et al. in 2016 [35] and Zhao et al. [36]. These meta-analyses concluded that colchicine could be beneficial in the prevention of POAF and post-ablation AF. A study that looked at the POAF and post-cardiac ablation/PVI separately was performed by Salih et al. in 2017 [37], and it concluded that colchicine had beneficial effects against AF in both groups. Lennerz et al. [38] concentrated their meta-analysis on POAF patients and found colchicine beneficial. However, a meta-analysis of POAF studies by Wang et al. in 2016 [39] found a neutral effect of colchicine against POAF. A review article by Deftereos et al. in 2019 [40] concluded that colchicine might have a preventive role in the development of AF after cardiac surgery or catheter ablation for PVI. Our meta-analysis includes the COCS trial [19], which was not included in the previous studies mentioned above. In addition, our meta-analysis includes four different sub-analyses, each analyzed separately rather than clubbing them together so that the results can be extrapolated correctly to the appropriate sub-group.

In post-cardiac-surgical patients, among the studies reviewed here, two studies showed a statistically significant benefit from colchicine, and four others showed a neutral effect. However, in our meta-analysis combining all these studies (Figure 2), we observe an overall benefit from colchicine in decreasing the incidence of POAF. In post-PVI/ ablation cases, individual studies reviewed in this article demonstrated mostly positive benefits against AF using colchicine. Our meta-analysis shows positive benefits of using colchicine against AF at three (Figure 3) and 12 months (Figure 4) in post-PVI/ablation patients.

Prior meta-analyses demonstrated similar benefits in both POAF and post-PVI/ablation groups; however, our meta-analysis differs in the following aspects. Ours adds a new study (the COCS trial) for the post-cardiac surgical group, not included in previous meta-analyses. Furthermore, we divided the patients in the post-PVI/ablation category into two different groups (at three months and 12 months), which helps identify the benefits at these different periods of follow up. Our study conducts a meta-analysis of colchicine's effect on patients with CAD as well (Figure 5). In patients with CAD, our study shows a neutral effect of colchicine in reducing the incidence of AF.

Our meta-analysis has certain limitations. The dosage, time of initiation, duration of administration of colchicine, and the follow-up times for recording AF differ across studies. Thus, the results and values derived in our meta-analyses are only approximate. The side effect incidence, including diarrhea, could be a limiting factor in the continued use of colchicine but is not analyzed within the scope of this meta-analysis. Of the two studies included in CAD, one of them dealt with patients with acute CAD (COLCOT [23]) and the other with chronic CAD (LoDoCo2 [24]). Hence, when interpreting the meta-analysis under the CAD category, one should be aware of that limitation. In the post-PVI/ablation category, patients with different LA-EAT volumes categorized in one of the studies (the 2015 study by Egami et al. [21]) are clubbed together in our meta-analysis, which may have affected our study results on post-PVI/ablation patients.

There is a need for further research to identify and confirm the benefits of colchicine against AF. On clinicaltrials.gov, we have identified four ongoing randomized controlled trials which are being undertaken specifically to see if colchicine has any benefits against AF in certain cardiac conditions. These trials are 1) Use of Colchicine to Decrease Atrial Fibrillation Recurrence After Ablation (ClinicalTrials.gov Identifier: NCT05459974); 2) COP-AF trial (The Prevention of Perioperative Atrial Fibrillation in Patients Undergoing Thoracic Surgery) (ClinicalTrials.gov Identifier: NCT03310125); 3) IMPROVE-PVI Pilot Trial (Impact of Short-course colchicine Versus Placebo After Pulmonary Vein Isolation) (ClinicalTrials.gov Identifier: NCT04160117); and 4) Co-STAR Trial (colchicine for Patients with Aortic Stenosis Undergoing Transcatheter Aortic Valve Replacement) (ClinicalTrials.gov Identifier: NCT04870424). Once we have these studies completed and published, the data from these studies can help more in understanding the therapeutic benefits of colchicine against AF. 

## Conclusions

Our study concludes that colchicine may be beneficial for decreasing the incidence of AF in post-cardiac surgery patients and post-pulmonary vein isolation (PVI)/ablation patients. It may not decrease the incidence of AF in patients with coronary artery disease. Further large-scale randomized controlled trials with larger study groups under each category might help to confirm the results of this study.
